# Healthcare provision for HIV co-infected tuberculosis patients in rural Zambia: an observational cohort study at primary care centers

**DOI:** 10.1186/1472-6963-13-397

**Published:** 2013-10-08

**Authors:** Shinsuke Miyano, Samba Muvuma, Naoko Ishikawa, Hiroyoshi Endo, Charles Msiska, Gardner Syakantu

**Affiliations:** 1 National Center for Global Health and Medicine, 1-21-1 Toyama, Shinjuku-ku, Tokyo 162-8655, Japan; 2 Japan International Cooperation Agency, Lusaka, Zambia; 3 Chongwe District Medical Office, Chongwe, Zambia; 4 Department of International Affairs and Tropical Medicine, Tokyo Women’s Medical University, Tokyo, Japan; 5 Department of Clinical Care and Diagnostic Service, Ministry of Health, Lusaka, Zambia

**Keywords:** Tuberculosis, HIV, Operational research, Primary health care, Zambia

## Abstract

**Background:**

Linkage of healthcare services for tuberculosis (TB) and human immunodeficiency virus (HIV) remains a major challenge in resource-limited settings. Our operational research aimed to evaluate the linkage between TB and HIV services in a rural area of Zambia, and to explore factors associated with the enrolment of TB/HIV co-infected patients in HIV care services.

**Methods:**

All TB patients newly diagnosed as HIV-positive in Chongwe district, Zambia between 2009 and 2010 were included. Data from TB registers and medical records were reviewed. Patient referral to HIV services and provision of antiretroviral therapy (ART) were further examined through HIV registers and records.

**Results:**

Of 621 patients (median age 33.0 years, female 42.4%) who started anti-TB treatment, clinic records indicated that 297 patients were newly diagnosed as HIV-positive, and 176 (59.3%) of these were referred to an ART clinic. Analysis of records at the ART clinic found that only 85 (28.6%) of TB/HIV patients had actually been enrolled in HIV care, of whom only 58 (68.2%) had commenced ART. Logistic regression analyses demonstrated the following factors associated with lower enrolment: “male” sex (aOR, 0.45; 95% CI 0.26-0.78), “previous TB treatment” (aOR, 0.29; 95% CI, 0.11-0.75), “registration at sites that did not provide ART services (non-ART site)” (aOR, 0.10; 95% CI, 0.01-0.77) and “death on TB treatment outcome (aOR, 0.20; 95% CI, 0.06-0.65). However, patient registration at TB clinics in 2010 was associated with markedly higher enrolment in HIV care as compared to registration in 2009 (aOR, 2.80; 95% CI, 1.53-5.12).

**Conclusions:**

HIV testing for TB patients has been successfully scaled up. However referrals of co-infected patients still remain a challenge due to poor linkage between TB and HIV healthcare services. Committed healthcare workers, a well-organized health services system and patient education are urgently required to ensure a higher rate of referral of TB/HIV co-infected patients for appropriate care.

## Background

Zambia has the thirteenth highest tuberculosis (TB) incidence rate [1] and the seventh highest human immunodeficiency virus (HIV) prevalence [2] in the world. The Zambian Ministry of Health has adopted the directly observed treatment short-course (DOTS), and achieved nationwide coverage since 2003. In terms of the HIV/AIDS program, Provider-Initiated HIV Testing and Counseling (PITC) services at TB clinics have been scaled up since 2006, and have been available at all TB clinics since 2008 [3]. In 2005, free antiretroviral therapy (ART) services were introduced in hospitals, which has further expanded into selected rural health centers (RHCs) since 2008 through the unique national Mobile ART Services program [4]. Under this program, a mobile ART team, comprising medical professionals from a district hospital, visits selected RHCs every 2 weeks and assists with ART services. This program has contributed to decentralizing ART services at the primary healthcare level. Although TB and HIV services have been successfully established in Zambia, the linkage between these services in operational settings at the primary healthcare level seems poor and needs to be evaluated further. This study examined the degree of the linkage between TB and HIV services to identify factors affecting the linkage at the primary healthcare level in a rural district of Zambia.

## Methods

Chongwe district is located 100 km from Lusaka, the capital city of Zambia. Approximately 240,000 people live in this district, and are largely scattered in rural areas. Primary healthcare services, including diagnosis and treatment of TB and diagnosis of HIV, are provided in all 24 RHCs, which are located strategically to cover the entire population. ART services are available at 13 of these RHCs through the national Mobile ART Services program (ART sites) and two hospitals through routine ART clinic services. The other 11 RHCs do not provide ART services, and only provide diagnosis of HIV (non-ART sites).

This study included all TB patients registered as receiving TB treatment and who were newly diagnosed as HIV-positive in all the RHCs in Chongwe district in 2009 and 2010. These patients were individually followed to confirm three steps of linkage between TB and HIV services; step 1) whether TB clinic referred them to ART clinic, step 2) whether they reached any ART clinic in the district and were enrolled in HIV care and step 3) whether they initiated ART. As for step 1, we reviewed the data collected through the “national TB treatment registers” and “TB treatment cards”. As for step 2 and 3, we matched these patients with their name, sex and birthday at all ART clinics in the district using the “national HIV care registers”, “HIV case records” and the “district electronic information system for HIV patients”. When the information of these three steps was not recorded on these sources properly, the interview for each patient was conducted to confirm these steps. The characteristics of patients enrolled and not enrolled in HIV care were compared to identify influential factors.

Values of patients’ characteristics were calculated as medians or proportions. Univariate analysis was conducted by chi-square test to determine the statistical significance between the groups, and described using crude odds ratio (cOR) and 95% CI. A p-value of less than 0.05 was considered statistically significant. Multiple logistic regression analysis was performed to explore the factors associated with enrolment in HIV care. The variables of which *p* value was less than 0.1 in the univariate analysis were entered into the multiple regression model. The model was described using adjusted OR (aOR) and 95% CI. All statistical analyses were performed using SPSS Statistics software program, version 20.0 (SPSS Inc., Chicago, IL, USA).

### Ethics statement

This study was approved by the University of Zambia Research Ethics Committee, Lusaka, Zambia (Reference number 022-11-09).

## Results

### Characteristics of registered TB patients

During the study period, 621 patients were registered for TB treatment in Chongwe district (Table 1). The median age was 33.0 years, 42.4% were female, 505 (81.3%) suffered from pulmonary TB, and 518 (83.4%) were diagnosed with TB for the first time. More patients were registered in 2010 (56.5%) than in 2009, and at ART sites (88.8%) than non-ART sites. In terms of HIV diagnosis, 549 (88.4%) were tested for HIV and 360 (65.4%) were HIV-positive.

**Table 1:** Characteristics of all TB patients in Chongwe district (N=621)

	***n *(%)**
**Age _median [IQR]**	33.0 [25.0-42.0]
**Female**	263 (42.4%)
**Site of disease**	
**Pulmonary TB (PTB)**	505 (81.3%)
**Extra-pulmonary TB (EPTB)**	116 (18.7%)
**Registration group**	
**New**	518 (83.4%)
**Previously treated**	92 (14.8%)
**Other**	11 (1.8%)
**Registration year**	
**2009**	270 (43.5%)
**2010**	351 (56.5%)
**Registration site**	
**ART sites (13 RHCs)**	552 (88.8%)
**Non-ART sites (11 RHCs)**	69 (11.2%)
**Tested for HIV**	549 (88.4%)
**HIV positive**	360 (65.4%)
**Newly diagnosed**	297 (82.5%)
**Previously diagnosed**	63 (17.5%)

### Linkage from TB clinic to HIV services (ART clinic)

**Figure 1:**
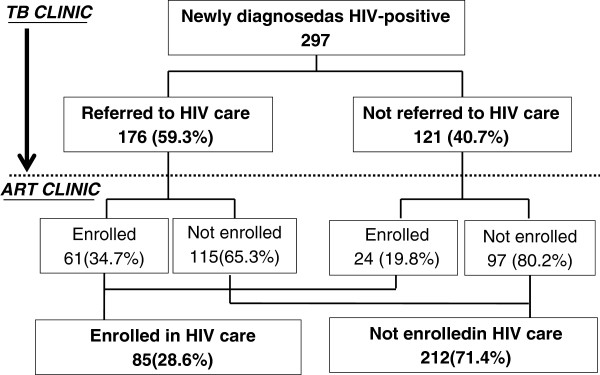
Linkage between TB and HIV services of newly diagnosed TB/HIV co-infected patients (N=297).

The TB clinic diagnosed 297 of the 360 TB/HIV patients as new HIV-positive patients (82.5%). Referral of these patients from the TB clinic to the ART clinic and their enrolment in HIV care at the ART clinic were followed up as shown in Figure 1. At the TB clinic, 176 (59.3%) of these patients were asked by healthcare workers to visit ART clinics for enrolment in HIV care, while the remainder (40.7%) were not asked.

Records of the ART clinics in the district were examined to trace these TB/HIV patients and to confirm whether they attended the ART clinic and enrolled in HIV care. Of those who were referred from the TB clinic, 61 (34.7%) were enrolled in HIV care. Of those who were not referred to ART clinics, 24 patients (19.8%) were enrolled in HIV care (*p* < 0.01). Thus, the overall enrolment rate of TB/HIV co-infected patients in HIV care was 28.6% (85/297).

### Factors associated with the enrolment of TB/HIV co-infected patients in HIV care

Factors associated with the enrolment of TB/HIV co-infected patients in HIV care were analyzed (Table 2). Statistically significant differences in univariate analysis were observed for sex, site of disease, registration group, registration year, registration site and death on TB treatment outcome between enrolled and non-enrolled patients. Logistic regression analyses demonstrated the following factors associated with lower enrolment: “male” sex (aOR, 0.45; 95% CI 0.26-0.78; *p* < 0.01), “previous TB treatment” (aOR, 0.29; 95% CI, 0.11-0.75; *p* < 0.01), “registration at sites that did not provide ART services (non-ART site)” (aOR, 0.10; 95% CI, 0.01-0.77; *p* = 0.03) and “death on TB treatment outcome (aOR, 0.20; 95% CI, 0.06-0.65; *p* < 0.01). However, patient registration in 2010 was associated with a significantly higher enrolment rate in HIV care as compared to registration in 2009 (aOR, 2.80; 95% CI, 1.53-5.12; *p* < 0.01).

**Table 2:** Factors analysis for the enrolment of TB/HIV co-infected patients in HIV care (N=297)

	**Enrolled (n=85)**	**Not enrolled (n=212)**	**cOR (95% CI)**	***p*-value**	**aOR (95% CI)**	***p*-value**
**Gender**						
**Female**	52 (61.1%)	91 (42.9%)	ref	**< 0.01**	ref	**< 0.01**
**Male**	33 (38.9%)	121 (57.1%)	0.49 (0.29-0.82)*		0.45 (0.26-0.78)*	
**Age group**						
**≤ 30**	36 (42.4%)	95 (44.8%)	ref	0.63		
**> 30**	48 (57.6%)	116 (55.2%)	1.14 (0.68-1.89)			
**Registration group**						
**New**	79 (92.9%)	169 (79.7%)	ref	**< 0.01**	ref	**< 0.01**
**Previously treated/Others**	6 (7.1%)	43 (20.3%)	0.30 (0.12-0.74)*		0.29 (0.11-0.75)*	
**Site of disease**						
**PTB**	61 (71.7%)	175 (82.5%)	ref	**0.03**		
**EPTB**	24 (28.3%)	37 (17.5%)	1.90 (1.05-3.44)*			
**Registration year**						
**2009**	28 (32.9%)	108 (50.9%)	ref	**< 0.01**	ref	**< 0.01**
**2010**	57 (67.1%)	104 (49.1%)	2.06 (1.21-3.49)*		2.80 (1.53-5.12)*	
**Registration site**						
**ARTsites**	84 (98.8%)	192 (90.6%)	ref	**0.01**	ref	**0.03**
**non-ART sites**	1 (1.2%)	20 (9.4%)	0.11 (0.02-0.87)*		0.10 (0.01-0.77)*	
**TB treatment outcomes**						
**Treatment success**	53 (62.3%)	107 (50.5%)	ref		ref	
**Died**	5 (5.9%)	29 (13.7%)	0.27 (0.01-0.80)*	**0.02**	0.20 (0.06-0.65)*	**< 0.01**
**Default**	22 (25.9%)	65 (30.1%)	0.68 (0.38-1.23)	0.20		
**Transfer out**	5 (5.9%)	11 (5.7%)	1.14 (0.30-2.78)	0.88		

*Statistically significant (*p* < 0.05).

### HIV care services for TB/HIV co-infected patients enrolled in HIV care

Of the 85 TB patients enrolled in HIV care, 76 (89.4%) were treated with co-trimoxazole preventive therapy, and 58 (68.2%) commenced ART within 12 months after registration for anti-TB treatment (Table 3). The median time between TB treatment registration and ART initiation was 54.5 days. Forty-eight patients commenced ART during TB treatment. In terms of the ART regimen, a nevirapine (NVP)-based regimen was prescribed significantly more often in patients whose TB information was not recorded on the ART patient records (*p* < 0.01).

**Table 3:** HIV care services for TB/HIV co-infected patients enrolled in HIV care (N=85)

	**n (%)**
**Co-trimoxazole (CPT) prophylaxis**	76 (89.4%)
**ART initiation**	58 (68.2%)
**Timing of ART initiation (days_median [IQR])**	54.5 [21.8-114.0]
**- During TB treatment (0–180 days)**	48 (82.8%)
**Initiation phase (0–60 days)**	33 (68.8%)
**Continuous phase (61-180 days)**	15 (31.2%)
**- After anti-TB treatment (> 180 days)**	10 (17.2%)
**ART regimen including Nevirapine (NVP) during TB treatment**	12 (22.6%)*
**TB information is recorded on ART patient’s record**	59 (70.2%)

* Statistically higher in patients whose TB information is not recorded on ART patient record (*p* < 0.01).

## Discussion

Our operational research showed that TB patients who were newly diagnosed as HIV-positive missed opportunities to be enrolled in HIV care. Firstly some TB patients who did not know their HIV status still missed the chance of testing although most patients had been successfully tested. Secondly many TB patients newly diagnosed as HIV-positive were not referred to ART clinic and not enrolled in HIV care properly. Lastly ART initiation and the ART regimen were also major issues even though these patients were enrolled in HIV care.

Once a patient is diagnosed with TB, HIV testing should be offered immediately if the HIV status is unknown. Other reports have already documented that integration of HIV and TB services improves the HIV testing rate among TB patients up to 94% in South Africa [5], 90% in Mozambique [6], 87% in Rwanda [7] and 91% in Malawi [8]. Our study also showed the similar percentage of HIV testing among TB patients. In Zambia the scale-up of the policy for PITC and the decentralization of HIV care services through the national mobile ART services program could have contributed to such a high HIV testing rate for TB patients. However, around one tenth of TB patients still did not get tested despite that TB patients had higher risk of HIV co-infection in high burden country like Zambia. Further investigations are required to clarify the reason why they do not get tested.

The national guidelines for ART and TB recommend that active TB patients co-infected with HIV should be promptly and effectively referred to HIV care [9,10]. However, referrals from TB to ART clinics were still inadequate in the primary healthcare setting. Furthermore, various factors such as male sex, history of previous TB treatment, registration site, registration year, and death on TB treatment outcome were identified as strongly associated with enrolment in HIV care. The guidelines also recommend that TB/HIV patients should commence ART according to CD4 cell count and patient condition, and that an efavirenz-based regimen is preferred to a NVP-based regimen. Although we found that most of the patients initiated ART during TB treatment, NVP-based regimen was still widely used for patients on TB treatment.

Operational implementation of TB/HIV integrated healthcare remains a major challenge in primary healthcare despite the availability of national guidelines for optimal TB/HIV co-treatments. Our analysis suggested that issues involving healthcare workers, the health services system, and patients were all implicated in the substandard treatment of many TB/HIV patients which resulted in the poor linkage between TB and HIV services.

### Healthcare workers

In some resource-limited settings, TB and ART clinics still function independently at the operational primary healthcare level [11,12], although some pilot projects have successfully integrated TB and HIV services [6,13-16]. There are three steps in operational settings for proper care of TB/HIV patients: 1) referral from the TB clinic; 2) attendance at an ART clinic and enrolment in HIV care; and 3) commencement of ART. Our results revealed that only 59.3% of TB/HIV co-infected patients (176/297) were referred from a TB clinic to an ART clinic, and 34.1% (85/297) were enrolled in HIV care, with only 16.2% (48/297) commencing ART during TB treatment. This suggests that healthcare workers at TB clinics might have been satisfied with only screening for HIV, thereby neglecting the referral of co-infected patients to ART clinics.

Pre-treatment loss to follow-up has been strongly associated with poor quality health services and minimal commitment on the part of healthcare workers [17,18]. The healthcare workers at the TB clinics may have mismanaged of providing options available for co-infected patients despite that all workers have already trained for TB/HIV care services. For example, a lack of sufficient information regarding the patients’ condition may have affected the results, such that patients may have chosen not to visit ART clinics and be enrolled in HIV care due to their lack of understanding of the disease and its treatment. Those patients who were not referred from a TB clinic showed a significantly lower enrolment rate in HIV care. The failure of all TB/HIV co-infected patients to be enrolled in HIV care may arise from poor service from healthcare workers, and would also affect the outcomes of these patients. Recent clinical trials have shown that earlier initiation of ART during TB treatment in patients co-infected with HIV significantly reduced mortality compared with patients initiating ART after TB treatment completion [19-22]. Death on TB treatment outcome was also associated with the enrolment in HIV care. The median days between the date of TB treatment initiation and the date of death in non-enrolled group was significantly shorter than that in enrolled group (31.0 vs. 86.5 days; *p* < 0.01) (not shown on the table). Some patients may have died before enrolment in HIV care. Patient’ earlier access to health facilities and earlier diagnosis and treatment might need to be more advocated among people in communities by health care workers to avoid these bad outcomes.

The linkage of patient information between TB and ART clinics also requires to be coordinated more effectively by healthcare workers at both clinics. Since TB and HIV registers and records were maintained separately at their respective clinics, and no information was exchanged directly, a lack of coordination may have influenced the low percentage of co-infected patients being enrolled in HIV care and receiving optimal treatment. In our study, the patients who were treated with the NVP-based regimen had a significantly higher percentage of their HIV case records missing TB treatment information. When healthcare workers at an ART clinic do not realize that the patient is receiving TB treatment, the NVP-based ART regimen may be given. This regimen has been shown to increase virological failure and mortality because of drug interactions between rifampicin (used to treat TB) and NVP [23]. Thus, sharing information between the two clinics would improve patients’ outcomes through healthcare workers providing appropriate monitoring and continuity of care to the patients.

### Health services system

Of the patients registered at RHCs, a significantly higher number of patients were enrolled in HIV care at the RHCs where ART services were available, although a small number of newly diagnosed HIV patients were enrolled in HIV care at non-ART sites.

Difficulties in transferring patients from a non-ART site to an ART site could explain the significantly lower enrolment at non-ART sites. It concurs with other studies which reported that the availability of one-stop services for TB and HIV at the same facility promoted the linkage between these two services [6,16,24]. In Zambia, decentralization of ART services through the national Mobile ART Services program, allowing HIV care delivery in areas without the services, was adopted as the national policy for one-stop services to strengthen linkage. However, even at ART sites, only 30.4% of the patients (84/276) were enrolled in HIV care. The clinic schedules, in which the TB clinic opens daily but the ART clinic opens every 2 weeks when the mobile ART services come, might also affect the enrolment of patients. Management of a patient’s appointments on the same day by both clinics requires strengthening to offer a one-stop service in terms of place and time.

Compared with patients registering at the TB clinics in 2009, there were twice as many patients registered in 2010 who were enrolled in HIV care. The activities of the TB/HIV coordinating committee organized by Chongwe District Medical Office at the end of 2009, likely contributed to this marked improvement. This committee has launched awareness campaigns for both healthcare workers and patients, and introduced a patient escort service to facilitate appropriate referrals between the two clinics at some RHCs. Not only national policy, but also local policy reflecting the operational situation, could play important roles in improve linkage of services especially in primary healthcare.

### Patients

Male sex and previous TB treatment were negatively correlated with enrolment in HIV care. No other studies have reported an association between these factors and enrolment in HIV care, though a number of studies have shown that male sex is associated with problems complying with ART. One study in South Africa reported an association between male sex with non-initiation of ART during TB treatment [25]. Others reported that men had higher early mortality on ART due to their presentation at a more advanced stage of disease [26,27], and a higher risk of ART treatment default [28]. These studies imply that males may have more apathy, a lack of insight and lower acceptance of not only ART but also HIV care. For example, in the real settings, men’s working schedule might have influenced their lower enrolment in HIV care. Working for their family would be more prioritized for them than going to the clinic.

We also found that patients with previous TB treatment were less likely to be enrolled in HIV care. Since some studies have reported that previous TB treatment is a risk factor for subsequent TB treatment default [29,30], it could also be a factor for defaulting in enrolment in HIV care. It might be related to attitudes among some group of cases called “defaulter personality”. Personal and/or social factors, such as financial burden and lack of social support, are important determinants of treatment adherence [31]. It might also affect the enrolment in HIV care among TB/ HIV co-infected patients. The previously treated TB patients also have to present to the TB clinic every day for streptomycin injections. A prolonged and more complex treatment with daily intramuscular injections coupled with more side effects and might increase the risk of quitting TB treatment and rejecting being enrolled in HIV care. However, the national guidelines for TB recommend that re-treatment must be administered under strict directly observed treatment during the entire therapeutic process [10]. These patients should have more opportunity to be enrolled in HIV care since they visit the health facility more often and are closely observed by healthcare workers. Failure in the enrolment in HIV care may also result from a lack of action by healthcare workers as already discussed above. Although the shortage of healthcare workers might affect the situation especially at the primary healthcare level, the proper referral protocol based on the national guidelines also requires to be reinforced through the re-fresher trainings.

### Limitations

This study has several limitations. First, there was a relatively small number included in the study. As decentralization of ART services through the national Mobile ART Services program were set up properly at selected RHCs in the middle of 2008, only information for 2009 and 2010 was available to evaluate the linkage between TB and HIV services at the primary healthcare level.

Patient information was obtained through routinely collected data in RHC registers. Matching of TB/HIV co-infected patients at ART clinic was done using the electronic information system for HIV patients. Although we tried to minimize missing or inaccurate data by asking all RHC staff to record and input patient data properly, some information bias in the data could be present. We may also have underestimated the number of enrolled patients, since our confirmation of enrolment in HIV care was done at ART clinics only within the district. However, maximum efforts to trace patients were made by RHC staff and community volunteers, which reflected the operational situation.

Although the sites for the mobile ART program rollout were selected randomly by the district medical office to cover as large an area as possible, ART sites had a greater burden of TB cases per site than non-ART sites. This could suggest the presence of a selection bias which might have affected our results. However there was no statistically significant difference between sites in the HIV testing rate (88.0% in ART sites, 91.3% in non-ART sites; *p* = 0.43) and the prevalence of newly diagnosed HIV (82.9% in ART sites, 77.7% in non-ART sites; *p* = 0.44).

## Conclusions

Although most TB patients were tested for HIV, testing was not carried out in approximately 12%. In addition, there were missed opportunities for many HIV-positive patients to be enrolled in HIV care. Several important factors were identified in the management of TB/HIV co-infected patients in Chongwe district which resulted in a failure to receive adequate treatment. Poor linkage between TB and HIV services could affect patient outcomes through not providing quality care. Although the health services system, supported by national and district policies, has recently strengthened the linkage between these services, in order to improve referral rates to ART clinics, enrolment in HIV care, and ART initiation in TB/HIV co-infected patients, greater commitment of healthcare workers to provide patient-centered services, and appropriate interventions for patients are urgently required. One-stop or partially integrated TB/HIV services [32] could be the next step to provide proper services. It should be established not only by making TB and ART clinics at the same place but also by managing patient’s clinic appointment date on her/his suitable one day and monitoring patients with one case record/register including her/his comprehensive information. Re-forming health services system in terms of “space”, “time” and “information” would be also helpful for healthcare workers to provide patient-centered services by assessing each patient more closely.

## Competing interests

We certify that there is no conflict of interest with any financial organization regarding the material discussed in this manuscript. This study was funded by Research Grant for International Health, H23-4, by the Ministry of Health, Labor and Welfare, Japan (http://www.ncgm.go.jp/kaihatsu/). The funders had no role in study design, data collection and analysis, decision to publish, or preparation of the manuscript.

## Authors’ contributions

SM1 and SM2 conceived and designed the study. SM1 collected and assembled the data. SM1 also analyzed and interpreted the data with SM2, NI and HE. All authors discussed the results and commented on the manuscript. All authors approved the final version of the article.

## Pre-publication history

The pre-publication history for this paper can be accessed here:

http://www.biomedcentral.com/1472-6963/13/397/prepub
